# Preparation and Characterization of Gelatin-Based Mucoadhesive Nanocomposites as Intravesical Gene Delivery Scaffolds

**DOI:** 10.1155/2014/473823

**Published:** 2014-12-15

**Authors:** Ching-Wen Liu, Li-Ching Chang, Kai-Jen Lin, Tsan-Jung Yu, Ching-Chung Tsai, Hao-Kuang Wang, Tong-Rong Tsai

**Affiliations:** ^1^School of Pharmacy, Kaohsiung Medical University, Kaohsiung 807, Taiwan; ^2^Department of Occupational Therapy, I-Shou University, Kaohsiung 824, Taiwan; ^3^Department of Pharmacy, E-DA Hospital, I-Shou University, Kaohsiung 824, Taiwan; ^4^Department of Pathology, E-DA Hospital, I-Shou University, Kaohsiung 824, Taiwan; ^5^Department of Urology, E-DA Hospital, I-Shou University, Kaohsiung 824, Taiwan; ^6^Department of Pediatrics, E-Da Hospital, I-Shou University, Kaohsiung 824, Taiwan; ^7^Department of Neurosurgery, E-Da Hospital, I-Shou University, Kaohsiung 824, Taiwan

## Abstract

This study aimed to develop optimal gelatin-based mucoadhesive nanocomposites as scaffolds for intravesical gene delivery to the urothelium. Hydrogels were prepared by chemically crosslinking gelatin A or B with glutaraldehyde. Physicochemical and delivery properties including hydration ratio, viscosity, size, yield, thermosensitivity, and enzymatic degradation were studied, and scanning electron microscopy (SEM) was carried out. The optimal hydrogels (H), composed of 15% gelatin A175, displayed an 81.5% yield rate, 87.1% hydration ratio, 42.9 Pa*·*s viscosity, and 125.8 nm particle size. The crosslinking density of the hydrogels was determined by performing pronase degradation and ninhydrin assays. *In vitro* lentivirus (LV) release studies involving p24 capsid protein analysis in 293T cells revealed that hydrogels containing lentivirus (H-LV) had a higher cumulative release than that observed for LV alone (3.7-, 2.3-, and 2.3-fold at days 1, 3, and 5, resp.). Lentivirus from lentivector constructed green fluorescent protein (GFP) was then entrapped in hydrogels (H-LV-GFP). H-LV-GFP showed enhanced gene delivery in AY-27 cells *in vitro* and to rat urothelium by intravesical instillation *in vivo*. Cystometrogram showed mucoadhesive H-LV reduced peak micturition and threshold pressure and increased bladder compliance. In this study, we successfully developed first optimal gelatin-based mucoadhesive nanocomposites as intravesical gene delivery scaffolds.

## 1. Introduction

Intravesical delivery is an administration method that consists of inserting a catheter from the urinary tract into the bladder cavity to deliver drugs. According to the 2013 European Association of Urology (EAU) guidelines for non-muscle-invasive bladder cancer (NMIBC), patients with a low- or intermediate-risk tumor should receive intravesical immunotherapy or instillations of chemotherapy [1]. The duration of drug instillation during intravesical bladder therapy is typically limited to 2 hours, after which the drug is drained out from the bladder. Sometimes chemotherapeutic drug-induced temporary irritative voiding will result in instillation of less than 2 hours. Under normal conditions, the six to seven cellular layers of the urothelium are almost impermeable to all irritants present in the urine; thus, intravesical administration must overcome the challenges posed by these barriers and urine flushing. Conventional vehicles used for intravesical delivery fail to provide sustained exposure to the drug inside the bladder, rarely lasting beyond the first voiding of urine after instillation. The low permeability of the urothelium and the low residence time of the drug in the bladder inevitably result in frequent instillation so as to avoid a high rate of recurrence [2, 3]. Thus, development of an optimal mucoadhesive drug or gene delivery scaffold would be a promising strategy for intravesical therapy.

Protein-based nanocarriers represent promising candidates for efficient drug and gene delivery owing to their low cytotoxicity, high drug binding capacity, and significant uptake into the targeted cells as well as the abundance of the renewable resources that can be used for their production [4, 5]. In gene delivery, protein nanoparticles can protect oligonucleotides from nuclease digestion and facilitate their transportation into the nucleus. Gelatin is a mixture of proteins obtained by acid or alkaline hydrolysis of collagen. It has excellent biocompatibility, biodegradability, and nonimmunogenicity and a great capacity for modification at the level of amino acids [6]. Gelatin types A and B are extracted from collagens by acidic and alkaline pretreatment, respectively. Alkaline pretreatment converts glutamine and asparagine residues into glutamic and aspartic acid, respectively, which increases the carboxylic acid content in gelatin type B to a greater extent than that in gelatin type A [7]. The functional groups of gelatin are accessible for various chemical modifications, which may be especially useful in developing targeted drug delivery vehicles [8]. Cationized gelatin nanoparticles have been reported as alternative carriers to existing DNA delivery systems [8].

A gelatin hydrogel can be formed by physical crosslinking in water; during the process, gelatin molecules aggregate and undergo a conformational change from a random coil to a triple helix [9]. However, the noncovalent associations are easily broken at temperatures higher than 30–35°C, which significantly limits their biomedical applications at the physiological temperature of 37°C. To increase its stability and mechanical properties, the gelatin gel can be covalently crosslinked by small chemicals such as carbodiimides, formaldehyde, and glutaraldehyde, which can couple the carboxyl groups with amino groups, forming stable amide bonds [10, 11]. The crosslinked gelatin can form an intricate high-molecular-weight network that is capable of swelling. This can act as a drug-filled matrix and drug depot owing to its bioadhesion to the bladder mucosa, thereby extending drug exposure in the bladder cavity beyond the voiding of urine [12–14]. Controlled mucoadhesive drug release after intravesical hydrogel administration will likely increased efficacy.

Gene delivery from hydrogel biomaterials provides a fundamental tool for a variety of clinical applications in regenerative medicine and in gene therapy for inherited disorders. The high water content and mild gelation conditions of hydrogels support their use for gene delivery by preserving the activity of lentiviral vectors and shielding vectors from any host immune response [15]. This biomaterial platform provides an opportunity to improve gene transfer by enhancing vector stability, while promoting and/or controlling cell-vector interactions in order to modulate the location and duration of transgene expression. Lentiviruses represent an attractive new vector system due to their ability to infect both nondividing and dividing cells, broad tropism, integration into the host genome which enables long-term availability of the encoded therapeutic protein, relative ease of production, and the availability of large libraries of constructs [15, 16]. Although some studies have addressed the crucial role of hydrogels in lentiviral gene delivery, to date, no study has evaluated the feasibility of optimized hydrogels as intravesical gene release scaffolds.

The major aim of our present study was to develop novel gelatin-based mucoadhesive nanocomposites and investigate their feasibility as scaffolds for intravesical gene release. These hydrogels were prepared from gelatins A (A75, A175) and B (B75, B225) by chemically crosslinking with glutaraldehyde. The physicochemical properties and thermosensitivity were then optimized for intravesical instillation. The crosslinking density was determined by performing a pronase degradation assay followed by ninhydrin-based assays. To verify the capacity of the hydrogels as scaffolds for gene delivery, pLKO_AS2, a lentiviral expression vector, was used as a model lentivector. The cumulative lentivirus (LV) release from the hydrogels was determined by measuring capsid protein p24. The transduction efficiency of LV constructed with the green fluorescent protein (GFP) gene (LV-GFP) was evaluated in bladder cancer AY-27 cells* in vitro* and rat urothelium* via* intravesical instillation* in vivo*. Subsequently, the urodynamic effects resulting from the hydrogels were also determined by obtaining cystometrograms (CMGs) in rats.

## 2. Materials and Methods

### 2.1. Materials

Gelatin, pronase, glycine, polybrene, and glutaraldehyde were purchased from Sigma Chemical Co. (St. Louis, MO). Deionized water was purified using a Milli-Q system (Millipore, Milford, MA, USA). All cell culture media and reagents were from Gibco BRL (Grand Island, NY, USA) or Hyclone (Logan, UT, USA). Viral particle concentrations were determined using HIV-1 p24 Antigen ELISA (ZeptoMetrix, Franklin, MA). Fischer F344 rats were purchased from the National Laboratory Animal Center (NLAC) (Taipei, Taiwan). Animal protocols were approved by the Animal Ethics Committee of I-Shou University.

### 2.2. Preparation of Gelatin Hydrogels

Hydrogels were prepared using types A and B gelatin with bloom numbers of 75, 175, or 225. A higher bloom number corresponds to a higher molecular weight of the polymer. An aqueous solution containing 5 wt.% gelatin (1 mL) with 0.8 *μ*g/mL of glutaraldehyde was left at 4°C overnight for gelation and cross-linking. The cross-linked gelatin hydrogels were immersed in a 50 mM glycine aqueous solution under agitation for 1 h to block the residual aldehyde groups of glutaraldehyde, followed by two washes in double-distilled water for 1 h. The resulting hydrogels were freeze-dried for 48 h. Yield represents the weight of the freeze-dried gelatin nanoparticles obtained after preparation, and this value is expressed as a percentage of the starting weight of gelatin [17].

### 2.3. Physicochemical Properties of Hydrogels

After allowing the freeze-dried hydrogels to swell for 24 h at 37°C in normal saline, the weight of swollen hydrogels (*W*_*s*_) was measured. The swollen hydrogels were dried in a vacuum drying oven at 60°C for 6 h, and then the weight of vacuum-dried hydrogels (*W*_*d*_) was measured. The hydration ratio was calculated using the following equation: [(*W*_*s*_ − *W*_*d*_)/*W*_*s*_] × 100 [3].

The viscosity of hydrogels was determined using a Carri-Med CSL2 100 rheometer (TA Instruments, USA). Particle size distribution and mean diameter were determined using an N5 Submicron Particle Size Analyzer (Beckman, USA) [18]. The surface morphology of freeze-dried hydrogels was determined using a field emission scanning electron microscope (FE-SEM) (JEOL JSM-5600 LV, Japan). Hydrogels were frozen in liquid nitrogen prior to freeze-drying to maintain the existing morphology. The sectioned gels were mounted on metal holders and vacuum coated with a gold layer prior to SEM examination [19].

The fluidity of the hydrogels was visually monitored at 25°C and 37°C. The absorption wavelengths of hydrogels were scanned using a DU-640 (Beckman Instruments, Fullerton, CA) spectrophotometer. The thermotransition of hydrogels was determined by measuring their absorption at 260 nm at 4, 26, 30, and 37°C.

To study the enhanced delivery to urothelium, hydrogels containing propidium iodide (PI) (500 *μ*L) were administered through a urethral catheter to a F344 rat bladder for 2 hours. The rat bladder was frozen, and specimens were embedded in Tissue-Tek O.C.T. compound (Miles Scientific, Elkhart, IN) and sectioned at 10 *μ*m thickness. The 4′,6-diamidino-2-phenylindole (DAPI) staining was performed to visualize the cell nuclei. Each sample was examined and evaluated under a fluorescence microscope (Eclipse 4000, Nikon, Tokyo, Japan).

### 2.4. Enzymatic Degradation of Hydrogels

The cross-linking density of hydrogels was determined by pronase degradation. Then, ninhydrin assays were used to quantify primary amine group formation after pronase treatment. Hydrogels were dispersed in PBS (pH 7.4) and incubated with pronase (0.5, 1, and 1.8 mg/mL). Hydrogel degradation was monitored by measuring the absorbance at 260 nm using a DU-640 spectrophotometer. The degradation percentage was calculated as *A*_*t*_/*A*_0_, where *A*_*t*_ is the absorbance at time *t* and *A*_0_ is the absorbance at time 0 [20]. After pronase treatment, protein release from the hydrogels was determined using the ninhydrin assay. When peptide bonds in gelatin hydrogels are cleaved due to the action of pronase, primary amines are formed. The concentration of amines was determined spectrophotometrically using ninhydrin at 570 nm with a DU-640 spectrophotometer, as described previously [21].

### 2.5. Lentivector Construction and Lentivirus Production

The cDNA expression lentivector (pLKO_AS2.puro, pLV) was obtained from the National RNAi Core Facility (Taiwan). Vectors containing GFP (pLV-GFP) served as an expression marker. Lentiviral particles were generated using standard molecular biology procedures [22]. Briefly, 293T packaging cells were seeded at 1.3 × 10^5^ cells/mL in 6 cm tissue culture plates containing DMEM supplemented with 10% FBS and antibiotics. After 24 hours, lentiviral packaging vectors [pCMV (8.2Δvpr), pMD.G (CMV-VSVG)] were co-transfected along with pLV into the 293T cells using TranslT-LT1 (Mirus, USA). After 18 hours, the medium was removed and replaced with fresh DMEM containing 10% FBS, and cells were cultured for another 24 hours. Then, the medium containing lentivirus (virus soup) was collected and used to infect target cells. Transduced cells were selected using puromycin, as described previously. The multiplicity of infection (MOI) was determined [23].

Freeze-dried hydrogels were dissolved in 1 mL of normal saline, and the solution was heated to 37°C for rat intravesical instillation. Five hundred microliters of virus soup (LV or LV-GFP) was dropped onto 500 *μ*L of 15% hydrogel for 10 min at 37°C.

### 2.6. Lentivirus-Associated HIV p24 Quantitation

The role of hydrogels as sustained release scaffold was performed by incubating hydrogels containing lentivirus with 293T cells in serum-containing media at 37°C for 0, 1, 3, and 5 days. Active infectious virus released from hydrogel resulted in more lentivirus particles produced from 293T cells, and lentivirus associated p24 protein can then be harvested from supernatant of culture medium. One hundred microliters of supernatant of culture medium was collected at the indicated time points and stored at −80°C until the sample concentration was determined. Hydrogels were degraded in pronase solution (Sigma Aldrich, St. Louis, MO, 1 mg/mL) to isolate the remaining virus from the gels. Viral particle concentrations were determined using an HIV-1 p24 Antigen ELISA (ZeptoMetrix, Franklin, MA) [24].

### 2.7. *In Vitro* Evaluation of Hydrogels Comprising LV-GFP

The rat bladder cancer cell line AY-27 (a gift from Professor R. Moore, University of Alberta, Canada) was cultured in RPMI-1640 medium containing 10% fetal bovine serum. AY-27 cells were treated with 15% gelatin A175 hydrogels (H), lentivirus (LV), hydrogels containing lentivirus (H-LV), LV-GFP, and hydrogels composed of LV-GFP (H-LV-GFP) for 18 h at an MOI of 3. Cell medium was removed and replaced with fresh DMEM containing 10% FBS, and cells were selected with puromycin (8 *μ*g/mL) and cultured for 10 days. DAPI was used to visualize cell nuclei along with GFP using a fluorescence microscope (Eclipse 4000, Nikon, Tokyo, Japan).

### 2.8. *In Vivo* Evaluation of Hydrogels Comprising LV-GFP

A urethral catheter was inserted into female F344 rats (7 weeks), and the rats were administered 500 *μ*L hydrogels containing LV or LV-GFP. The rats were transduced at an MOI of 10 for 2 hours, drained, and washed with normal saline on days 1, 3, and 5. On day 7, rat bladders were collected and frozen specimens were embedded in Tissue-Tek O.C.T. compound (Miles Scientific, Elkhart, IN). DAPI staining was performed to visualize cell nuclei. Each sample was examined and evaluated using a fluorescence microscope (Eclipse 4000, Nikon, Tokyo, Japan).

### 2.9. Cytotoxic Assay

Cell viability was determined using the CellTiter 96 Aqueous nonradioactive cell proliferation assay (MTS) according to the manufacturer's instructions (Promega, Madison). AY-27 cells (1 × 10^4^) were seeded in 96-well plates and treated with serial dilutions of H-LV-GFP for 18 h. Cytotoxicity is expressed as the mean ± S.D. of four experiments [18].

### 2.10. Cystometrogram (CMG) and Data Analysis

The CMGs were carried out according to the method previously described [25]. In brief, in each experiment, Fischer F344 rats (7 weeks) were anesthetized with Zoletil-50 (1 mg/kg intraperitoneal injection injection). Before the beginning of each CMG, the bladder was emptied and a urethral catheter was indwelled and used to fill the bladder and to measure bladder pressure. The catheter was connected via a T-tube to a syringe pump (KDS250, KDScientific Corp., MA, USA), pressure transducer and amplifier (ML866 and ML224, PowerLab, ADInstruments, CO, USA), recorded on a chart recorder, and digitized for computer data collection (Labchart 7, ADI Instruments: Windows 7 system). Then the bladder was infused with 500 *μ*L normal saline (as control) and 15% gelatin A175 hydrogels (H) at a steady rate (0.07 mL/min), during which the pressure was measured in-line with the catheter.

A voiding contraction was defined as an increase in bladder pressure that resulted in urine loss. CMG was recorded until the bladder pressure was stable and at least 5 filling/voiding cycles were measured on each rat before drug administration and used as baseline values. CMG parameters recorded for each animal included peak micturition pressure, threshold pressure, duration of nonvoiding contractions (without urine leakage during bladder infusion), and bladder compliance. Peak micturition pressure was the maximum pressure during micturition as observed in CMG. Threshold pressure was the intravesical pressure right before the initiation of micturition. Bladder compliance was measured by infused volume (*μ*L)/threshold pressure (ΔcmH_2_O) [26].

### 2.11. Statistics

Data were expressed as the mean ± SD. The two-sided Student's *t*-test was used to determine differences between groups. A *P* value < 0.05 was considered significantly different.

## 3. Results

### 3.1. Characterization and Thermosensitivity Analysis of Hydrogels

We initially characterized the impact of gelatin type on hydrogel yield, hydration ratio, viscosity, and size (Table 1). In our production method for glutaraldehyde-crosslinked hydrogels, a high molecular weight (as well as a high bloom number) of gelatin produced a high yield ratio. For gelatin types A75, A175, B75, and B225, the mean yield ratios were 75.2, 81.5, 53.0, and 60.0%, respectively. In eight formulations (5% and 15% of four gelatin items), the hydration ratios and particle sizes ranged from 86.3 to 94.9% and 110.6 and 179.5 nm, respectively. The study aimed to develop nanonized hydrogels for intravesical gene delivery. The mucoadhesive property of hydrogels is crucial to protect against urine flush in the bladder and provide scaffolds for sustained LV release. Therefore, gelatin A175 was selected for the following physical, chemical, and biological assays based on its high viscosity, optimal yield, and hydration ratio.

**Figure 1:**
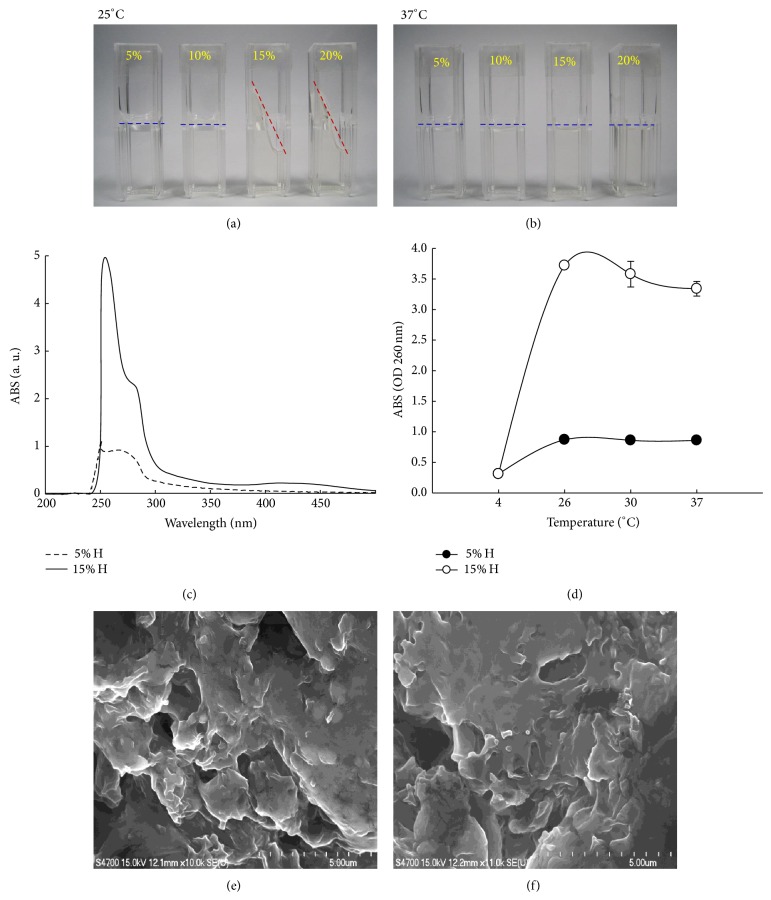
Characterization of hydrogels. Visual inspection of 5%, 10%, 15%, and 20% A175 hydrogels at (a) 25°C and (b) 37°C. (c) Wavelength scans of 5% and 15% hydrogels. (d) Thermotransitional phase exchange was measured at 4, 26, 30, and 37°C and absorbance was measured at 260 nm. FE-SEM of freeze-dried (e) 5% and (f) 15% hydrogels (magnification, ×10000).

First, we examined the thermosensitivity (fluidity and turbidity) of 5, 10, 15, and 20% A175 hydrogels at 25°C and 37°C (Figures 1(a) and 1(b)). The 15% A175 hydrogels showed high viscosity at 25°C and became fluid at 37°C. Achieving optimal mucoadhesion and fluidity to cover the urothelium when instilled into the bladder may afford sustained gene delivery after intravesical instillation. The appearance of both 5 and 15% A175 hydrogels are transparent at 25°C, however, their viscosities being 2.6 and 42.9 Pa*·*s, respectively.

Thermotransition measurements confirmed the thermosensitivity of the A175 hydrogels. The highest absorption wavelength observed for the 5 and 15% A175 hydrogels was approximately 260 nm, as determined by scanning spectra from 200 to 500 nm (Figure 1(c)). Absorption measurements at 260 nm at 4, 26, 30, and 37°C revealed that the 15% A175 hydrogels demonstrated the highest thermosensitivity (Figure 1(d)). The field emission scanning electron microscopy (FE-SEM) images of the 5 and 15% A175 hydrogels showed gelatin crosslinked with glutaraldehyde (Figures 1(e) and 1(f)). Fifteen percent hydrogels had a more conglomerate and less porous structure.

The potential cytotoxicity of hydrogels in AY-27 cells was examined. The survival ratios after treatment with 0, 1.2, 2.5, 5, 10, and 20 *μ*g/mL hydrogels were 100, 98.3 ± 3.3, 96.5 ± 2.2, 96.5 ± 3.7, 96.9 ± 3.2, and 97.3 ± 4.0%, respectively.

### 3.2. Enhanced Mucoadhesion of Hydrogels by* In Vivo* Intravesical Instillation

**Figure 2:**
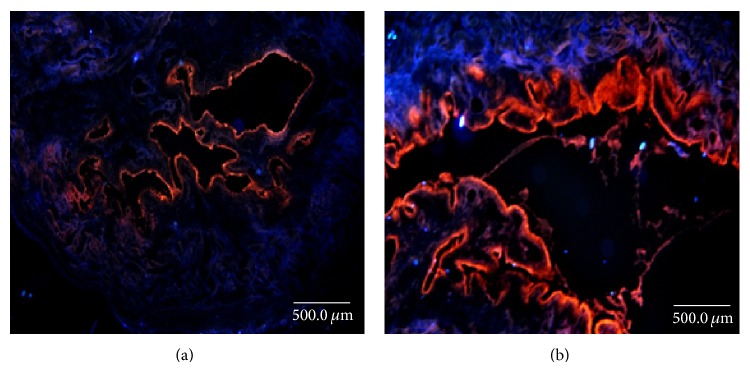
Fluorescence images of rat urothelium tissues after* in vivo* intravesical instillation of (a) 5% and (b) 15% hydrogels containing propidium iodide (PI, red). F344 rats were intravesical administered through a PE50 urethral catheter for 2 hours instillation. The rat bladder was harvested, embedded, and sectioned at 10 *μ*m thickness. Samples were examined under a fluorescence microscope (Eclipse 4000, Nikon, Tokyo, Japan) (magnification ×40).

To confirm the potential of hydrogels for entrapping and delivering chemical drugs* in vivo*, 5 and 15% A175 hydrogels containing propidium iodide (PI) were introduced into F344 rat bladders* via* a 2-hour intravesical instillation. The PI content in the urothelium was observed as red fluorescence under a fluorescent microscope. The 5% hydrogels (Figure 2(a)) only delivery PI to the surface cells (also called umbrella or dome cells) of the urothelium, whereas the 15% hydrogels (Figure 2(b)) transduced PI to the umbrella, intermediate, and basal cells of the urothelium, reaching the subepithelial connective tissue of the bladder. Thus, the highly viscous 15% A175 hydrogels displayed greater mucoadhesion and PI delivery than did the 5% hydrogels.

### 3.3. Enzymatic Degradation of Nanonized Gelatin Hydrogels

**Figure 3:**
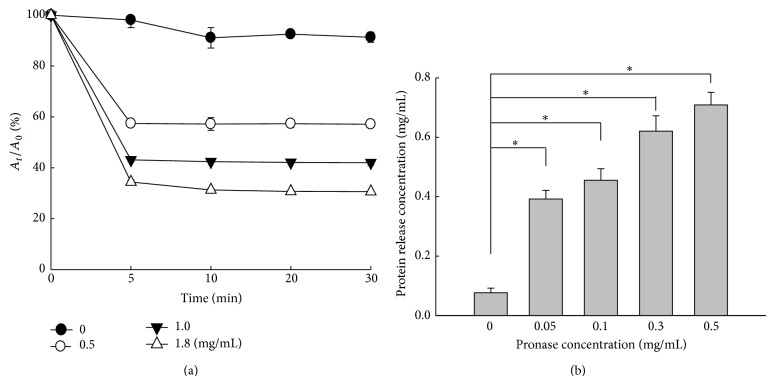
Enzyme degradation of hydrogels. (a) Hydrogels were incubated with 0, 0.5, 1.0, and 1.8 mg/mL pronase at 37°C and absorbance at 260 nm. *A*_*t*_, absorbance at time *t*. *A*_0_, absorbance at time 0. (b) Protein release from the hydrogels after pronase degradation was measured using ninhydrin assays (*n* = 3, ^*^ *P* < 0.05).

Protease (e.g., pronase) degradation of the hydrogels was performed to determine their crosslinking density. The 15% A175 hydrogels demonstrated biphasic kinetics of degradation in a dose-dependent manner (Figure 3(a)). Pronase induced rapid degradation within 5 min, and the rate of degradation slowed for the next 25 min. In response to increasing pronase concentrations (0.5 to 1.8 mg/mL), hydrogels degraded in a concentration-dependent manner. The enzymatic degradation of gelatin nanoparticles is consistent with the degradation of glutaraldehyde crosslinkers by proteolytic enzymes [20].

Pronase-mediated degradation of hydrogels results in primary amine group formation, which can be measured using the ninhydrin assay. In response to pronase (0, 0.05, 0.1, 0.3, and 0.5 mg/mL) treatment, the mean protein release from the hydrogels was 0.08, 0.32, 0.46, 0.62, and 0.71 mg/mL, respectively (Figure 3(b)). Therefore, increased pronase promoted hydrogel degradation in a dose-dependent manner, increasing protein release due to the hydrolysis of amide bonds. Optimal viscosity, mucoadhesiveness, size, yield, and thermosensitivity were achieved using the 15% gelatin A175 hydrogels (H); thus, this formulation was used for subsequent LV entrapment and intravesical gene delivery studies.

### 3.4. Hydrogels as Scaffolds for LV Release

**Figure 4:**
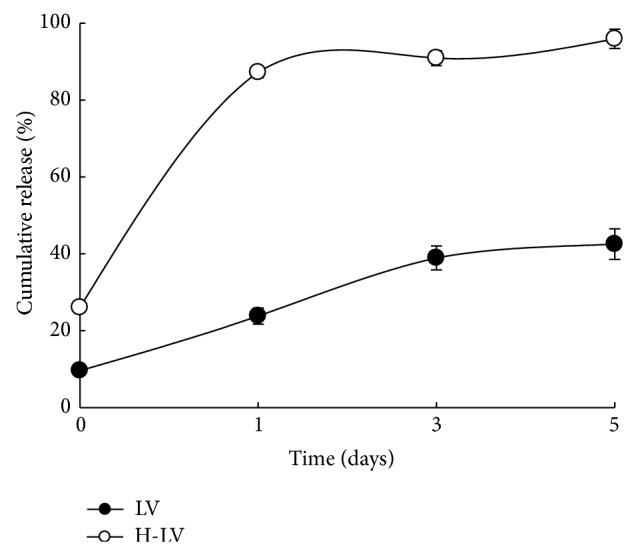
Evaluation of cumulative lentivirus release from hydrogels entrapping lentivirus (H-LV) or lentivirus (LV) alone in 293T cells by measuring HIV-p24 content (*n* = 3). The conditioned medium was harvested 24 hours after infection. The amount of p24 in supernatant was measured by p24 ELISA.

The ability of 15% A175 hydrogels to serve as scaffolds for LV release was determined by measuring the HIV-p24 content, an indicator of lentiviral titers. Lentivirus (LV) alone and 15% A175 hydrogels (H) containing LV (H-LV) were individually transfected into 293T cells. As shown in Figure 4, H-LV produced significantly higher cumulative and sustained release of the p24 capsid protein than LV alone. The cumulative releases of LV/H-LV were 9.6/26.0% initially (2.7-fold), 23.8/87.2% at day 1 (3.7-fold), 38.9/90.9% at day 3 (2.3-fold), and 42.5/95.9% at day 5 (2.3-fold). This cumulative release profile confirms the utility of 15% A175 hydrogels (H) as potential scaffolds for LV release.

### 3.5. Hydrogels Enhanced Lentiviral Gene Delivery* In Vitro*

**Figure 5:**
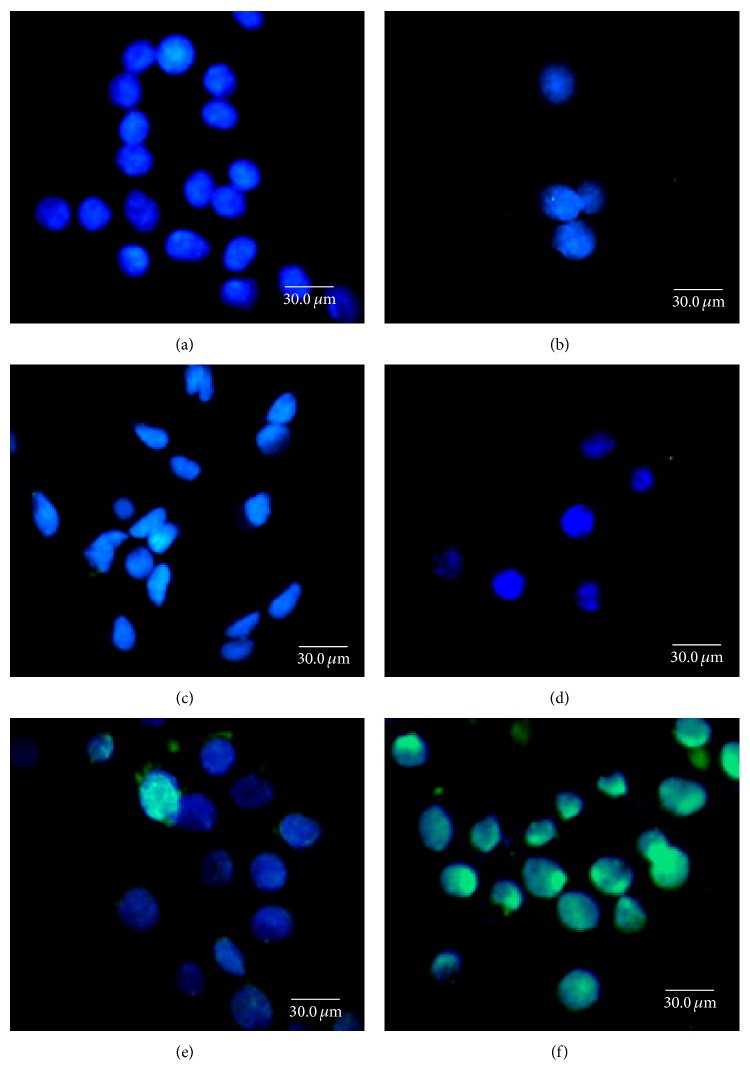
Fluorescence images of AY-27 cells at day 10 after transfecting with hydrogel entrapped LV-GFP. AY-27 rat bladder cancer cells (a) were treated with (b) hydrogels (H), (c) lentivirus (LV), (d) hydrogels containing lentivirus (H-LV), (e) LV constructed with the GFP gene (LV-GFP), and (f) hydrogels containing LV-GFP (H-LV-GFP). Cells expressing GFP appear green while cell nuclei stained with DAPI appear blue (magnification ×200).

To evaluate the potential of hydrogels for enhancing lentivirus delivery, LV and LV-GFP were to infect AY-27 bladder cancer cells. As shown in Figure 5, LV-GFP displayed low transduction efficiency in AY-27 cells (Figure 5(e)). However, LV-GFP entrapped in 15% A175 hydrogels (H-LV-GFP) demonstrated markedly higher GFP expression, indicative of enhanced gene delivery* in vitro*. In contrast, AY-27 cells (Figure 5(a)) transduced with hydrogels (Figure 5(b)), LV (Figure 5(c)), and H-LV did not express GFP, indicating that the entrapped complexes have no GFP bioactivity to measure. Thus, lentiviruses entrapped in hydrogels may enhance gene delivery and expression* in vitro*.

### 3.6. Hydrogels Enhanced Lentiviral Gene Delivery to the Rat Urothelium* In Vivo*

**Figure 6:**
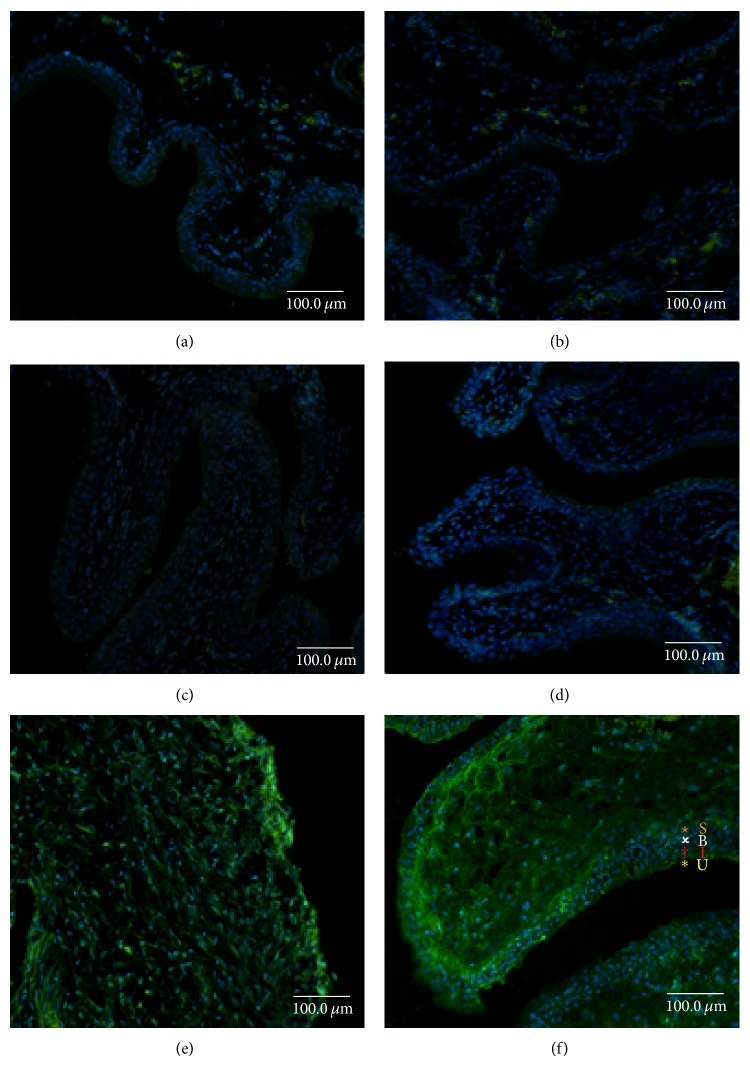
Fluorescence images of F344 rat urothelium at day 7 after intravesical instillation with hydrogel entrapped LV-GFP. Fluorescence images revealed the normal urothelium (a) after treatment with (b) H, (c) LV, (d) H-LV, (e) LV-GFP, and (f) H-LV-GFP. The urothelium (U: umbrella cells; I: intermediate cells; B: basal cells; S: subepithelial connective tissue) was counter-stained with DAPI (nuclei, blue; magnification ×40).

Next, we evaluated the gene delivery efficiency of lentivirus entrapped in gelatin hydrogels to the rat urothelium by intravesical instillation. On the seventh day after LV-GFP instillation, GFP was only expressed in the top one to two layers of umbrella cells of the urothelium (Figure 6(e)). However, in response to H-LV-GFP, GFP was expressed in all of the layers of the urothelium (umbrella, intermediate, and basal cells), reaching the subepithelial connective tissue (Figure 6(f)). In contrast, no GFP expression was observed in the control urothelium (Figure 6(a)) or with hydrogel (Figure 6(b)), LV (Figure 6(c)), or H-LV (Figure 6(d)). The cell nuclei were counter-stained with DAPI (blue). These data confirm those observed with hydrogels containing PI, suggesting that hydrogels can enhance chemical or LV delivery to the urothelium by intravesical instillation in the rat bladder* in vivo*.

### 3.7. Effects of Hydrogels on Cystometric Parameters

**Figure 7:**
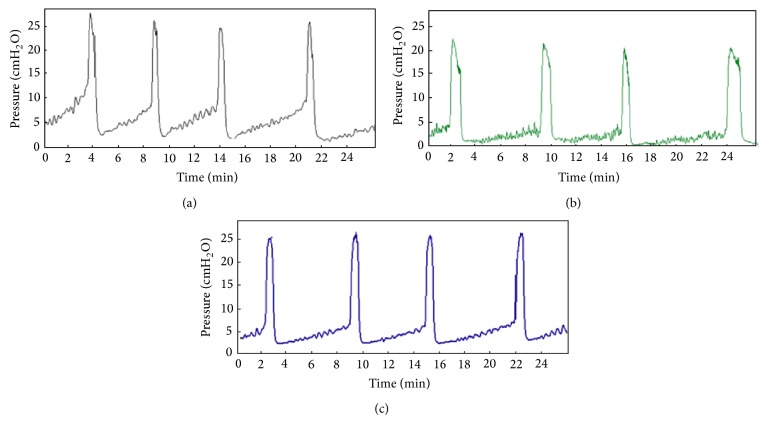
Representative continuous cystometrogram (CMG) recordings in F344 rats treated with intravesical (a) normal saline in control and (b) LV, (c) H-LV. The CMG recorded using continuous intravesical infusion of saline (0.7 mL/min) on anesthetized rats.

CMGs are shown in Figure 7 and the recorded CMG parameters of peak micturition pressure, threshold pressure, micturition duration, and bladder compliance are shown in Table 2. In voiding contractions, peak micturition pressure (cmH_2_O) showed significant decrease in LV (14.8 ± 0.4) and H-LV (18.7 ± 0.6) groups compared to that in the saline (23.0 ± 2.5, *P* < 0.05). Threshold pressure (cmH_2_O), defined as intravesical pressure right before the initiation of micturition, also significantly decreased in the LV (2.4 ± 0.3) and H-LV (4.8 ± 0.2) groups compared to that in the saline (4.8 ± 0.2 versus 9.1 ± 0.6, *P* < 0.01). There was no significant intergroup difference in micturition duration, indicating that hydrogels may not irritate the bladder nor induce bladder hyperactivity. Bladder compliance, measured by infused volume (*μ*L)/threshold pressure (ΔcmH_2_O), was significantly higher in LV (29.3 ± 3.5) and H-LV (14.7 ± 0.7) groups compared to that in the saline (7.7 ± 0.5, *P* < 0.05). These results indicated that mucoadhesive hydrogels decreased micturition maximum pressure and threshold pressure, but increased bladder compliance.

## 4. Discussion

This paper describes the first successful use of modified thermosensitive gelatin-based nanocomposites as a suitable matrix for localized and sustained gene delivery to the urothelium* via *the intravesical route. Intravesical therapy provides the advantage of selectively delivering drugs in high concentrations to the tumor-bearing bladder while minimizing systemic exposure [20]. The formation of hydrogels using varied concentrations and Bloom numbers of gelatin A and gelatin B allowed for a range of physical and chemical properties and localized release of gene therapy vectors to the urothelium. Hydrogels were prepared by the chemical crosslinking method to form glutaraldehyde-modified gelatin nanocomposites. Hydrogels with 15% gelatin A175 were the optimal mucoadhesive scaffolds for releasing entrapped LV for intravesical usage. With regard to the cumulative LV release, hydrogels with entrapped lentiviruses showed higher sustained release for up to 5 days by intravesical instillation.

In the present study, we used glutaraldehyde as a chemical crosslinking agent to produce 125.8 nm nanonized gelatin hydrogels. We initially loaded PI, which mimics chemical drugs, into the hydrogels to verify that the hydrogels enhanced PI delivery to the urothelium. The higher viscosity of the 15% gelatin A175 hydrogels afforded greater mucoadhesion to the urothelium, showing resistance against urine flushing and resulting in higher penetration of the PI to the urothelium. Lee et al. [27] evaluated the absorption of paclitaxel from intravesically administered glyceryl monooleate (GMO) containing paclitaxel in a rabbit model. The GMO/paclitaxel/water 70% group produced a 10.5-fold higher tissue concentration than did the taxol group. Lu et al. [20] prepared paclitaxel-loaded gelatin nanoparticles (gelatin A175) using the desolvation method to produce particles with sizes ranging from 600 to 1000 nm. These paclitaxel-loaded nanoparticles were active against human RT4 bladder transitional cancer cells; moreover, the drug concentrations in the urothelium and lamina propria tissue layers, where Ta and T1 tumors are located, were 2.6-fold higher than those after intravesical instillation of paclitaxel in dogs. These previous studies showed that gelatin is a promising carrier for intravesical instillation of chemical drugs, but not as an intravesical gene delivery scaffold.

Gelatin hydrogels usually dissolve into a colloidal sol at temperatures above 37°C in water, and they congeal into gels at lower temperatures (e.g., room temperature); thus, gelatin is not frequently considered as a candidate material for tissue engineering applications [28]. In this study, we optimized gelatin hydrogel formulations to achieve the desired viscosity and release for use in intravesical instillation. Our formulations for producing hydrogels provide optimal fluidity at room temperature, are easy to transduce through a catheter into the bladder, and show mucoadhesion when coating the urothelium inside the bladder.

*In vivo* gene transfer is a feasible approach for the treatment of superficial bladder cancer because of its specific features, such as easy external access for treatment through the urethra and a low risk of gene transfer to other organs [29]. However, the bladder permeability barrier (BPB) for intravesical drug delivery restricts the penetration of chemical drugs and transgene expression. A bioadhesive drug delivery system may overcome the limitation of drug retention time and benefit LV-mediated gene delivery. Wood et al. [30] reported that intravesical administration of replication-incompetent adenovirus was successful in restricting transgene expression only to the bladder for 2 days after administration, and expression was reduced severalfold by the third day. In the present study, we successfully demonstrated that hydrogel entrapped LV-GFP may afford sustained expression of the GFP protein in the urothelium for up to 7 days. Replication-incompetent HIV-1-based lentiviral vectors have emerged as powerful, safe tools for gene delivery because of their ability to transduce dividing and nondividing cells [31]. This enables the integration of genes into the host genome to sustain stable transgene expression, and pseudotyping with vesicular stomatitis virus glycoprotein (VSV-G) or other modified envelope proteins can aid in efficient transduction of the cells of interest [32]. The hydrogels prepared in this study demonstrated their efficiency as scaffolds and reservoirs for lentiviruses that are resistant to urine voiding and enabled sustained gene expression. However, further safety studies of gelatin-entrapped LV following intravesical instillation are needed before it becomes a clinically feasible drug delivery system.

*In vivo* strategies based on retaining the vector within the hydrogels must maintain the vector's function until the cells infiltrate the hydrogels [22]. Our study showed the MOIs of LV-GFP and H-LV-GFP measured in AY-27 cells were 6.86 and 8.20, respectively (data not shown). This indicated the loading of hydrogels on LV may increase the transduction efficiency. Kidd et al. [24] developed subcutaneous fibrin gels containing virus or hydroxylapatite (HA)/virus and implanted them into male CD-1 mice. The ability of the hydrogels to promote gene delivery* in vivo* was significant, because expression levels increased steadily from day 3 to day 9, with maximal expression at day 9. This model used subcutaneous administration of LV-loaded hydrogels. However, intravesical drug or gene delivery has to overcome its own set of challenges, the most prominent of which is the low residence time of a drug in the bladder, which necessitates frequent instillation. Conventional vehicles used for intravesical delivery fail to provide sustained exposure of drug inside the bladder, rarely lasting beyond the first voiding of urine after instillation [2]. Thus, hydrogels for lentiviral gene delivery should be considered for intravesical instillation. Our* in vivo* studies suggest that hydrogels containing LV provide at least 1 week of sustained gene expression and show high transduction efficiency to all of the urothelium layers (umbrella, intermediate, and basal cells), reaching the subepithelial connective tissue.

Although the bonding strength of the formaldehyde crosslinking was satisfactory, the impact of the hydrogels on urodynamic parameters was further studied. The high viscosity of the optimized 15% gelatin A175 hydrogels produced high mucoadhesion that helped protect the entrapped LV from urine voiding. These hydrogels did not significantly change the voiding duration; instead, the hydrogels reduced peak micturition pressure and threshold pressure.

One limitation of the present study is the fact that gelatin-based mucoadhesive nanocomposites may seem as suitable intravesical gene delivery scaffolds; however, the enhanced lentivirus transduction and sustained gene expression may also arise the safety concerns. Thus, deliberate immunotoxic evaluation of this hydrogels is suggested for future application in bladder cancer gene therapy.

## 5. Conclusion

In summary, we investigated a promising gelatin hydrogel delivery system as LV release scaffold to promote gene delivery into the urothelium. Enhanced lentiviral retention and penetration from the hydrogels produced more localized, efficient* in vivo* intravesical transgene expression. The gelatin hydrogels developed in this study have broad applications for gene therapy of bladder diseases.

## Figures and Tables

**Table 1:** Characterization of gelatin hydrogels.

Item	Gelatin type	Bloom number^a^	Yield (%)^b^	Concentration (wt%)	Hydration ratio (%)^b^	Viscosity (Pa*·*s)^b^	Particle size (nm)^b^
A75	A	75	75.2 ± 3.7	5	94.1 ± 0.93	3.4 ± 0.0	177.0 ± 4.9
				15	86.9 ± 0.1	16.6 ± 1.3	179.5 ± 6.9

A175	A	175	81.5 ± 10.5	5	94.9 ± 0.19	2.6 ± 0.0	135.2 ± 5.8
				15	87.1 ± 0.1	42.9 ± 0.6	125.8 ± 7.4

B75	B	75	53.0 ± 1.6	5	93.8 ± 0.17	1.3 ± 0.0	117.9 ± 1.1
				15	86.3 ± 0.4	15.4 ± 1.3	135.7 ± 1.3

B225	B	225	60.0 ± 7.0	5	94.9 ± 0.6	1.9 ± 0.6	117.9 ± 7.4
				15	87.4 ± 0.1	36.5 ± 0.6	110.6 ± 11.9

^a^The bloom number is proportional to the average molecular weight.

^b^Data are mean ± SD (*n* = 3).

**Table 2:** Comparison of the urodynamic parameters between control and H-LV.

Items	Saline	LV	H-LV	*P *		
				Saline/LV	Saline/H-LV	LV/H-LV
Peak micturition pressure (cmH_2_O)	23.0 ± 2.5	14.8 ± 0.4	18.7 ± 0.6	0.007^*^	0.037^*^	<0.001^*^
Threshold pressure (cmH_2_O)	9.1 ± 0.6	2.4 ± 0.3	4.8 ± 0.2	<0.001^*^	<0.001^*^	<0.001^*^
Duration (s)	368.0 ± 26.0	370.0 ± 47.7	332.5 ± 16.9	0.946	0.095	0.220
Bladder compliance (*μ*L/cmH_2_O)	7.7 ± 0.5	29.3 ± 3.5	14.7 ± 0.7	0.008^*^	<0.001^*^	0.002^*^

Data are mean ± SD. The symbol ∗ indicates statistical significance at *P* < 0.05.
